# COCONUT online: Collection of Open Natural Products database

**DOI:** 10.1186/s13321-020-00478-9

**Published:** 2021-01-10

**Authors:** Maria Sorokina, Peter Merseburger, Kohulan Rajan, Mehmet Aziz Yirik, Christoph Steinbeck

**Affiliations:** Institute for Inorganic and Analytical Chemistry, University Friedrich-Schiller, Lessing Strasse 8, 07743 Jena, Germany

**Keywords:** Natural products, Database, NoSQL, MongoDB, Molecular similarity search, Molecular substructure search

## Abstract

Natural products (NPs) are small molecules produced by living organisms with potential applications in pharmacology and other industries as many of them are bioactive. This potential raised great interest in NP research around the world and in different application fields, therefore, over the years a multiplication of generalistic and thematic NP databases has been observed. However, there is, at this moment, no online resource regrouping all known NPs in just one place, which would greatly simplify NPs research and allow computational screening and other *in silico* applications. In this manuscript we present the online version of the COlleCtion of Open Natural prodUcTs (COCONUT): an aggregated dataset of elucidated and predicted NPs collected from open sources and a web interface to browse, search and easily and quickly download NPs. COCONUT web is freely available at https://coconut.naturalproducts.net.

## Introduction

Natural products (NPs) have received constant attention from the scientific community due to their relevance in drug discovery, chemical ecology and molecular biology in general. In a recently published review on NPs databases [1] we inventoried over 120 natural products databases that have been published and used in the last 20 years. However, 16% of these are not available online anymore, 40% are commercial and their content cannot be easily accessed. The open resources are generally either specialized on a particular type of NPs, either lack annotations. For instance, the catalog of NPs from the ZINC database [2] is composed of over 80,000 entries, some of which can be purchased, but apart from their structure and that they are from natural origin, no additional information is provided. Super Natural II [3] is considered as the largest among all the NP databases, is accessible online in 2020, but it seems not to be maintained anymore and is mainly composed of compounds that can be purchased. Another recent database, NPAtlas [4], is constantly growing and extremely well annotated, but it is focusing on microbial NPs only. Another major NPs category, plant-produced compounds, also called phytochemicals, is available in several popular and well maintained databases, such as NuBBEDB [5], KnapSack [6], CMAUP [7] and TCM@Taiwan [8]. In addition to these relatively big databases, there is a plethora of smaller, more specialized NPs collections, such as FooDB [9], a user-friendly database hosting a relatively large number of NPs that are found in food. There is, therefore, a need for a generalistic NPs database, that will efficiently aggregate NPs information from various sources, improve its annotation and offer a pleasant user experience. With this ultimate goal in mind, we first assembled the most complete up-to-date COlleCtion of Open Natural ProdUcTs (COCONUT) that we have been continuously curating and annotating. Studies [10, 11] showed that fragments from NPs present in COCONUT have high diversity and structural complexity, which makes it, among other possible applications, a suitable source for drug discovery and can be included in drug design pipelines. Our next step was to make this data available to the scientific community as a full-fledged online natural products database, maintained at https://coconut.naturalproducts.net.

The COCONUT database is free and open to all users and there is no login required to access it. Its web interface allows diverse simple searches (e.g. by molecule name, InChI, InChI key, SMILES, drawn structure, molecular formula), advanced search by molecular features, together with substructure and similarity searches. Users can also download the whole dataset or search results in different formats. The database can be queried programmatically via a REST API, which facilitates COCONUT integration in workflows. The web interface, the back-end and the database are deployed as Docker containers, making it easily portable for hosting other sets of NPs and to be deployed on local installations.

**Fig. 1:**
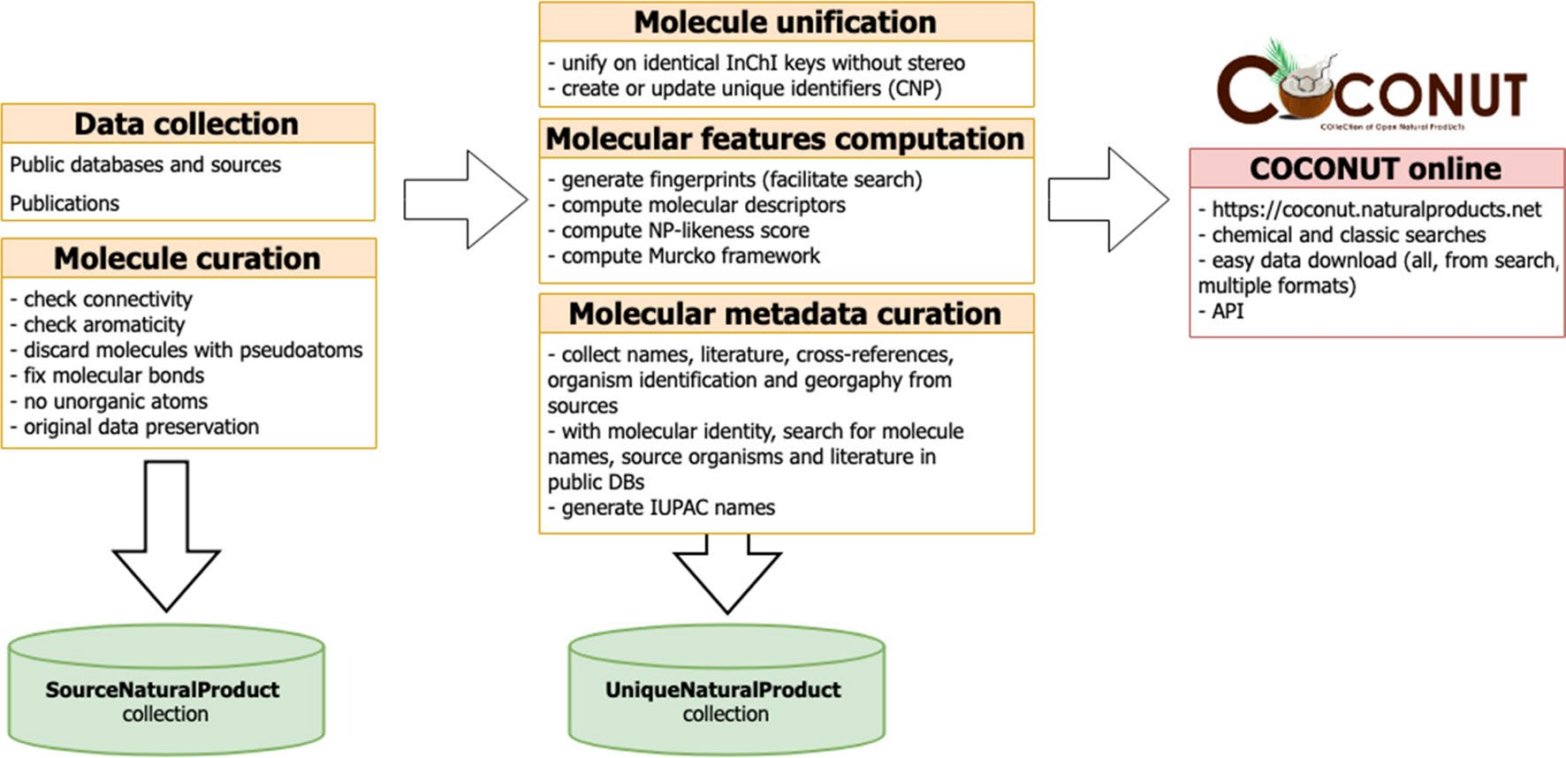
Construction and curation of COCONUT online

## Construction and content

COCONUT is assembled from a large number of chemical data sources (Table 1), from which NPs have been thoroughly extracted, curated, processed and annotated. The resulting NPs collection is presented within the full-fledged chemical database developed specially for this purpose (Fig. 1). Data curation and processing steps, together with the construction of the web interface and the description of available features are described below.

**Table 1:** Public databases and datasets from which COCONUT was assembled

Database name ("NP" suffix is added to database name when only a subset of it contains natural products)	Number of entries integrated in COCONUT from the source	Most resent publication or resource URL
AfroCancer	365	[33]
AfroDB	874	[34]
AfroMalariaDB	252	[35]
AnalytiCon Discovery NPs	4908	[36]
BIOFACQUIM	400	[37]
BitterDB	625	[38]
Carotenoids Database	986	[39]
ChEBI NPs	14603	[20]
ChEMBL NPs	1585	[21]
ChemSpider NPs	9027	[40]
CMAUP (cCollective molecular activities of useful plants)	20868	[7]
ConMedNP	2504	[41]
ETM (Ethiopian Traditional Medicine) DB	1633	[42]
Exposome-explorer	478	[43]
FooDB	22123	[9]
GNPS (Global Natural Products Social Molecular Networking)	6740	[44]
HIM (Herbal Ingredients in-vivo Metabolism database)	962	[45]
HIT (Herbal Ingredients Targets)	470	[46]
Indofine Chemical Company	46	[47]
InflamNat	536	[48]
InPACdb	122	[49]
InterBioScreen Ltd	67291	[50]
KNApSaCK	44422	[6]
Lichen Database	1453	[51]
Marine Natural Products	11880	[52]
Mitishamba database	1010	[53]
NANPDB (Natural Products from Northern African Sources)	3914	[54]
NCI DTP data	404	[55]
NPACT	1453	[56]
NPASS	27424	[57]
NPAtlas	23914	[4]
NPCARE	1362	[58]
NPEdia	16166	[59]
NuBBEDB	2022	[5]
p-ANAPL	467	[60]
Phenol-explorer	681	[61]
PubChem NPs	2828	[27]
ReSpect	699	[62]
SANCDB	592	[63]
Seaweed Metabolite Database (SWMD)	348	[64]
Specs Natural Products	745	[65]
Spektraris NMR	242	[66]
StreptomeDB	6058	[67]
Super Natural II	214420	[3]
TCMDB@Taiwan (Traditional Chinese Medicine database)	50862	[8]
TCMID (Traditional Chinese Medicine Integrated Database)	10552	[68]
TIPdb (database of Taiwan indigenous plants)	7742	[69]
TPPT (Toxic Plants–PhytoToxins)	1483	[70]
UEFS (Natural Products Databse of the UEFS)	481	[71]
UNPD (Universal Natural Products Database)	156865	[72]
VietHerb	4759	[73]
ZINC NP	67327	[74]
Manually selected molecules	61	x

### Data provenance, model and content

COCONUT data has been extracted from 53 various data sources and several manually collected from literature sets, as shown in Table 1. In the current COCONUT release (October 2020), there are 406,076 unique “flat” (with no stereochemistry) NPs, and a total of 730,441 NPs where stereochemistry has been preserved when available.

Every molecule collected from external sources passed a quality control and a registration procedure, where its structure is checked for size (between 5 and 210 heavy atoms), connectivity (only the biggest connected structure is kept), presence of pseudo-atoms, if implicit and explicit hydrogens are correct, and if the bonds are correct and the valences are conserved. The Kekulé representation is also assigned to the aromatic systems of each compound. At this step, tautomers and ionisation states are standardized following the ChEMBL chemical structure curation pipeline [12].

Then, NPs from different provenance are unified based on the identity of their InChI keys without stereochemistry. This unification step is performed without stereochemistry, as in different data sources stereochemistry is not systematically present and can also be represented differently. When available, the original molecular structure with stereochemistry is preserved and can be accessed for each NP entry.

The authors are well aware that different stereoisomers of a compound can have very different biological activity. The procedure described above was a necessary step to create a unified resource out of distributed databases of varying quality. Further curation will gradually improve stereochemical assignments and linkage to original source articles.

Each unique NP is then assigned a unique identifier, composed of the “CNP” prefix and 7 digits. An automatic curation for NP metadata is performed, comprising the retrieval of its official name, synonyms, cross-references to other major chemical databases. Then, a range of molecular properties, descriptors and fingerprints (full list in Table 2) are computed using the in-build CDK [13] libraries. As the number of the computed properties is quite big (73 fields in each document corresponding to one unique NP), only a selected fraction of them is displayed on the COCONUT web interface. Finally, the first round of automatic curation of NP metadata, in particular the molecular name synonyms, cross-references with other major chemical databases, correction of the literature references (PubMed identifiers and DOIs) and taxonomy is performed. All original data, unified NPs and the derived and calculated information are stored in MongoDB. The chemical classification of all NPs in COCONUT is performed with ClassyFire [14], and, when available, is displayed in the corresponding section of the compound page. ClassyFire provides a hierarchical chemical classification of chemical compounds and enables grouping NPs by their chemical class. Additionally, frameworks facilitating NPs analyses for their chemical and therapeutic properties are computed for NPs, such as Murcko frameworks [15], Ertl Functional Groups [16] and deepSMILES [17]. DeepSMILES is an adaptation of SMILES for use in deep machine learning of chemical structures. Due to the increased usage of deep learning in chemistry, it is indeed interesting to provide this new chemical representation type pre-computed for NPs.

Last, the annotation level of each NP in COCONUT is computed. It is a 5-star-based system, where 1 star is the lowest annotation quality (no verified common name, no taxonomic provenance annotation, no literature reference and no trusted data source) and 5 stars is the highest quality, with all the intermediate annotation qualities reflected by 2, 3 and 4 stars. Only ChEBI [18], KNApSAcK [6], ChEMBL [19], CMAUP [7], NPAtlas [4] and, of course, the manually picked data are considered as trusted data provenances. For example, caryolivine (CNP0235854) has a 5-stars annotation because it has a verified common name, is known to be produced by *Caryomene olivascens*, a plant, is associated to a scientific publication and is present in KNApSAcK. The COCONUT NP CNP0330764 has no verified common name, only a computed IUPAC one, but is present in CMAUP and is known to be produced by a range of plants, therefore it’s annotation level is 3. The annotation level is represented with stars on the NP page.

**Table 2:** Molecular features present in COCONUT and in their disponibility in the web interface

Natural product feature	Field name in MongoDB—uniqueNaturalProduct collection	Displayed on the website
COCONUT identifier	coconut_id	x
List of SMILES with stereochemistry and their provenance	absolute_smiles	x
AlogP (Ghose-Crippen LogKow)	alogp	x
AlogP2	alogp2	x
AMR—molar refractivity	amralogp	
Annotation level of the NP (from 1 to 5)	annotationLevel	x
BCUT decriptor (Eigenvalue based)	bcutDescriptor	
Bond number in the NP	bond_count	x
BPol descriptor	bpol	x
CAS number	cas	x
List of literature DOIs mentioning the NP	citationDOI	x
Boolean—if the molecule contains linear sugars	contains_linear_sugars	
Boolean—if the molecule contains circular sugars	contains_ring_sugars	
Boolean—if the molecule contains sugar moieties	contains_sugar	
deepSMILES	deep_smiles	x
Eccentric Connectivity Index Descriptor	eccentricConnectivityIndexDescriptor	x
List of tl Functional Groups	ertlFuntionalFragments	
List of Ertl Functional Groups in pseudo SMILES	ertlFunctionalFragmentsPseudoSmiles	
FMF descriptor	fmfDescriptor	x
List of data sources containing the NP	found_in_databases	
Fragment complexity descriptor	fragmentComplexityDescriptor	x
List of circular fragments (molecular signatures) of the deglycosylated NP	fragments	
List of circular fragments (molecular signatures) of the whole NP	fragmentsWithSugar	
Fractional CSP3 Descriptor (non-flatness of a molecule)	fsp3	x
List of continents and regions where the organism producing the NP is found	geoLocation	
Gravitational index descriptor (heavy atoms only)	gravitationalIndexHeavyAtoms	
Hydrogen bond acceptor count	hBondAcceptorCount	
Hydrogen bond donor count	hBondDonorCount	
Number of heavy atoms in the NP	heavy_atom_number	x
Hybridization Ratio Descriptor (fraction of sp3 carbons to sp2 carbons)	hybridizationRatioDescriptor	
InChI (without stereochemistry)	inchi	x
InChI key	inchikey	x
IUPAC name	iupac_name	x
First kappa shape index	kappaShapeIndex1	
Second kappa shape index	kappaShapeIndex2	
Third kappa shape index	kappaShapeIndex3	
Number of failures in the Lipinski rule of 5	lipinskiRuleOf5Failures	x
LogP descriptor (Mannhold version)	manholdlogp	
Maximal number of rings in the NP	max_number_of_rings	x
Minimal number of rings in the NP	min_number_of_rings	x
Molecular formula	molecular_formula	x
Molecular weight	molecular_weight	x
Murcko Framework	murcko_framework	x
Official name (when available)	name	x
NP-likeness score	npl_score	x
NP-likeness score computed on the glycosylated molecule	npl_sugar_score	
Total number of carbons	number_of_carbons	x
Total number of nitrogens	number_of_nitrogens	
Total number of oxygens	number_of_oxygens	
Number of sporo atoms	numberSpiroAtoms	
Petitjean Number Descriptor	petitjeanNumber	x
Petitjean geometrical shape index	petitjeanShapeGeom	
Petitjean geometrical shape index	petitjeanShapeTopo	
PubChem fingerprint in MongoDB BinData format	pubchemBits	
PubChem fingerprint as list of booleans	pubchemFingerprint	
SMILES with all hydrogen explicit	smiles	x
Number of heavy atoms of the deglycosylated moiety	sugar_free_heavy_atom_number	
SILES of the deglycosylated moiety	sugar_free_smiles	
Total atom number of the deglycosylated moiety	sugar_free_total_atom_number	
List of synonym names of the NP	synonyms	x
List of NCBI taxonomy identifiers of organisms producing the NP	taxid	
List of organisms producing the NP in text form	textTaxa	
Topological polar surface area descriptor	topoPSA	
Total atom count in the NP (incuding hydrogens)	total_atom_number	
Fractional polar surface area descriptor	tpsaEfficiency	
Unique SMILES (CDK)	unique_smiles	x
Volume descriptor	vabcDescriptor	
Vertex adjacency information	vertexAdjMagnitude	
Wiener Path Number	wienerPathNumber	x
Wiener Polarity Number	wienerPolarityNumber	
XLogP descriptor	xlogp	x
Cross-references to toher chemical ressources	xrefs	x
Zagreb Index	zagrebIndex	x
Chemical superclass of the NP computed with ClassyFire	chemicalSuperClass	x
Chemical class of the NP computed with ClassyFire	chemicalClass	x
Chemical subclass of the NP computed with ClassyFire	chemicalSubClass	x
Direct parent in the chemical ontology of the NP computed with ClassyFire	directParentClassification	x

#### Natural product naming

NPs common names in COCONUT have been retrieved, when available, from their databases of origin. The remaining NPs have been searched by InChI in major chemical databases (PubChem, ChEMBL and ChEBI) and common names and synonyms were retrieved when occurences the compound were found. Additionally to this, IUPAC names were computed for all COCONUT NPs using ChemAxon’s MolCovert [20], to add nomenclature homogeneity to the dataset. Furthemore, the IUPAC names are used as the main NP name when no official chemical name has been found nor in the original sources, nor by searching big chemical databases. Therefore, all NPs in COCONUT have at least one molecular name.

#### Computed molecular features

**Fig. 2:**
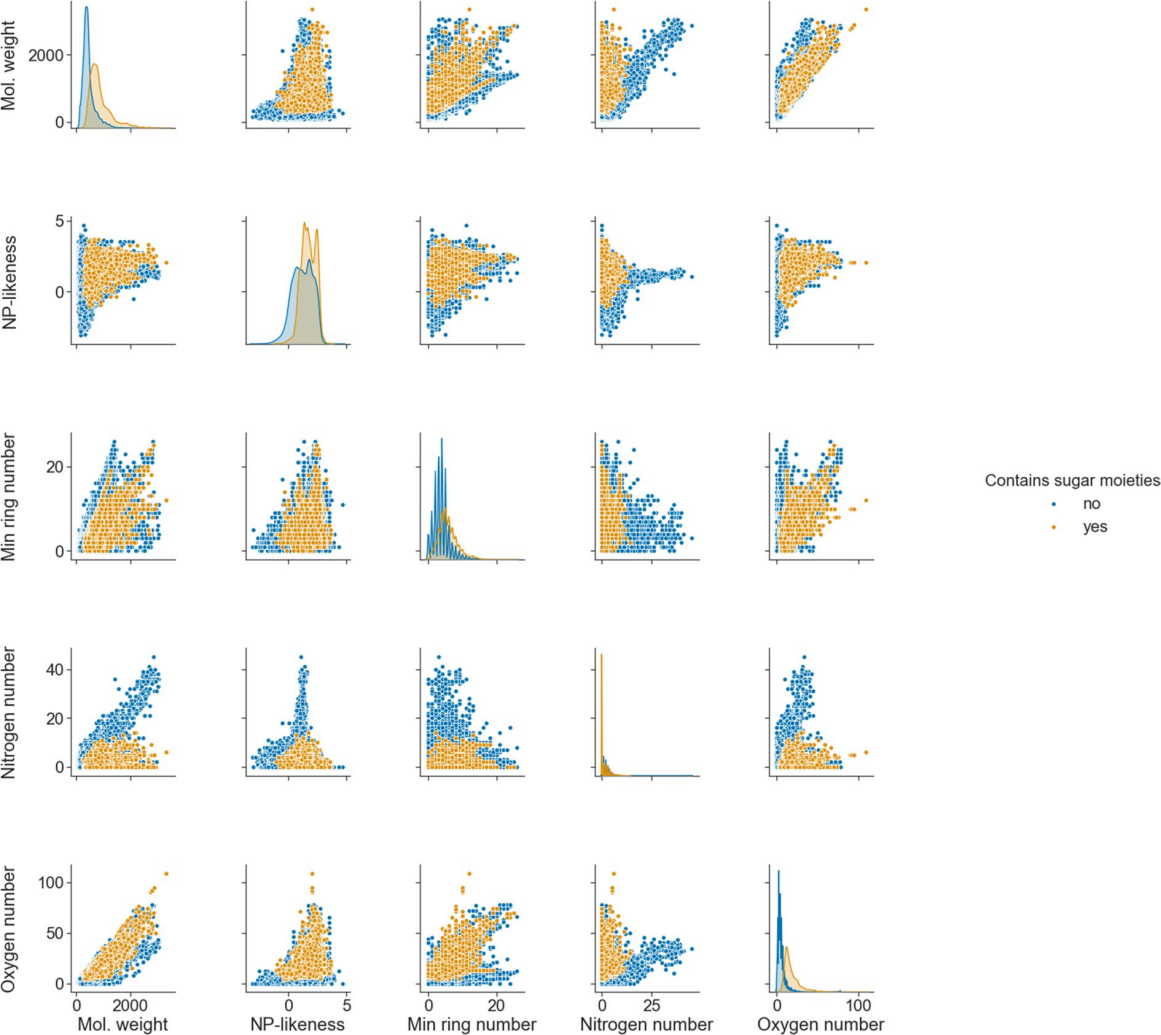
Pair plot of a selection of five of the molecular features available in COCONUT. Colour mapping corresponds to the presence (yellow) and absence (blue) of glycosidic moieties in the molecular structures of the NPs in COCONUT

Figure 2 demonstrates the distributions and relationships of a small selection of computed molecular features within COCONUT. Sugar moieties occur frequently in NPs and have an important impact on their bioactivities and physicochemical properties. However, they are often redundant and therefore obstruct the study of the aglycon. For this reason, NPs in COCONUT have been analysed for sugar moieties presence, and a deglycosylated structure representation was made available in the database. Sugar moieties manipulations were performed using the Sugar Removal Utility [21]. To track their influence on other features, their absence and presence are colour-mapped (no sugar moiety in the molecular structure in blue, and the presence of at least one sugar moiety in orange). The wide molecular weight range is typical for NPs; it is, however, interesting to notice its correlation with the number of oxygen atoms in the molecule, regardless of the presence and absence of sugar. Another interesting correlation to be noted is between the molecular weight and the nitrogen atom number in sugar-free molecules. The NP-likeness score [22], trained on high-quality NPs dataset and computed with NaPLeS [23], which was trained on high-quality NPs dataset, has a typical distribution for an NPs set, where most molecules have a positive score.

At this point, an additional NPs curation step has been performed, due to the possible inconsistency in genuine NPs of one of the used sources, SuperNatural II. NPs that are not occuring in other datasets used to assemble COCONUT, but only in SuperNatural II, have been thoroughly tested. To be kept in COCONUT and be considered as a genuine or predicted NP, such a molecule has to have a strictly positive NP-likeness score, be classified as a NP by NPclassifier [24], a deep neural network-based structural classification tool specialised in NPs or have a sugar moiety in its structure. The 24,880 molecules from SuperNatural II that didn’t pass this additional quality control have been removed from COCONUT, until further proof of their natural provenance.

Counting rings in a molecule can become a complex task, as the outer perimeter of two fused rings can be counted as one big ring. With more condensed rings, the number of fused ring perimeters (aka as the set of all rings) can grow steeply. In Fig. 2, only the minimal ring count (the minimal cycle base) is represented.

#### Natural product annotation

The particularity of NPs, opposed to synthetic molecules, which constitute the biggest part of chemical databases, resides in their production by living organisms. Therefore, in addition to their structure and computable structural properties, NPs need to be annotated with at least one literature reference, mentioning where, when and from which organism the NP was isolated. As a direct consequence, an NP entry should be associated with at least one organism, preferentially with an NCBI taxonomy identifier and the geographic location where the organism is naturally occuring or has been collected. Regrettably, this metadata is often omitted in public databases from which COCONUT was assembled. Therefore, only 31.5% (134,379) of NPs in COCONUT are annotated with at least one organism taxa, for 15.4% (66,068) of NPs the geographic location (on the continent level) of the organism occurence or collection is known and only 16.6% (70,730) of NPs have at least one literature reference. These numbers combine both the retrieval of the original NP annotations from their sources and our efforts to retrieve more extensive information from major trusted chemical databases, PubChem [25], ChEMBL [19], ChEBI [18], CMAUP [7] and KnapSacK [6]. Despite our efforts, most of the links between the original publication of the structure elucidation of an NP and its reference, source organism and its geographical location are still missing. A possible solution to fill these gaps is manual curation, but the amount of data in COCONUT is redhibitory for even considering this approach. Another solution is to use unsupervised machine learning for optical recognition approaches, to parse modern peer-reviewed literature and books to re-establish links between NPs structures and their provenance.

**Fig. 3:**
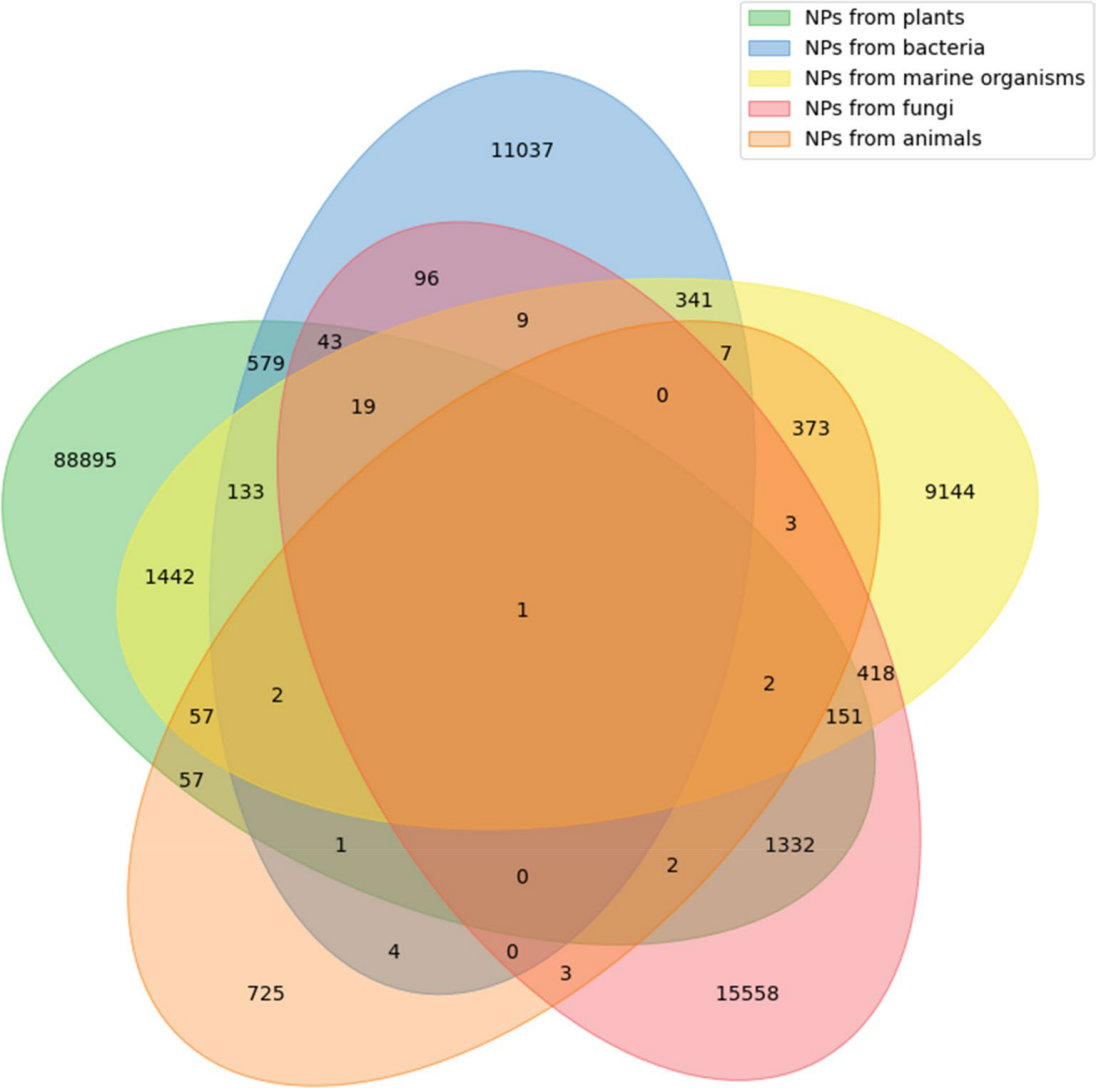
Overlap of NPs taxonomic provenance in COCONUT

We analysed the taxonomic classification of known NPs producers together with overlaps in NPs production between superkingdom for the 31% of the NPs in COCONUT for which the provenance organism is known (Fig. 3). Here are distinguished five taxonomic categories: plants, bacteria, fungi, animals and marine. The last one is not a proper monoclade classification, but rather reflects a group of organisms that are found only in marine and oceanic environments, and therefore can overlap in terms of its species and NP content with other categories, which are more stringent taxonomically. A large part (65%) of these annotated NPs are produced only by plants, and only very few (0.5%) are from animal origin. Main overlaps in terms of NP production between the taxonomic kingdoms are between plants and marine organisms (which is unsurprising, as there can be real plants among the marine entities) and surprisingly between plants and fungi. The other overlaps between taxonomic kingdoms are not as significant. It needs to be pointed out here that multicellular organisms, such as plants, animals and some of the fungi are most of the time in symbiosis with microorganisms, in particular bacteria. Therefore, NPs isolated from such a multicellular organism can be synthesized and secreted by their symbionts or microbiomes, and therefore mistakenly assigned to an incorrect organism.

The geographic location of the collection or the natural presence of the NP-producing organism is a piece of information that is even more difficult to obtain. Nowadays, a range of organisms, and in particular plants, can be found in different parts of the planet due to globalisation and their success in human consumption (e.g. garlic, tomatoes, curcuma or ginger). It is, therefore, difficult, if not impossible, to determine their original provenance. Also, the geographical information is often omitted in literature and most NPs databases. When available, the geographical provenance is stored in the MongoDB dump of COCONUT, but not displayed on the website.

**Fig. 4:**
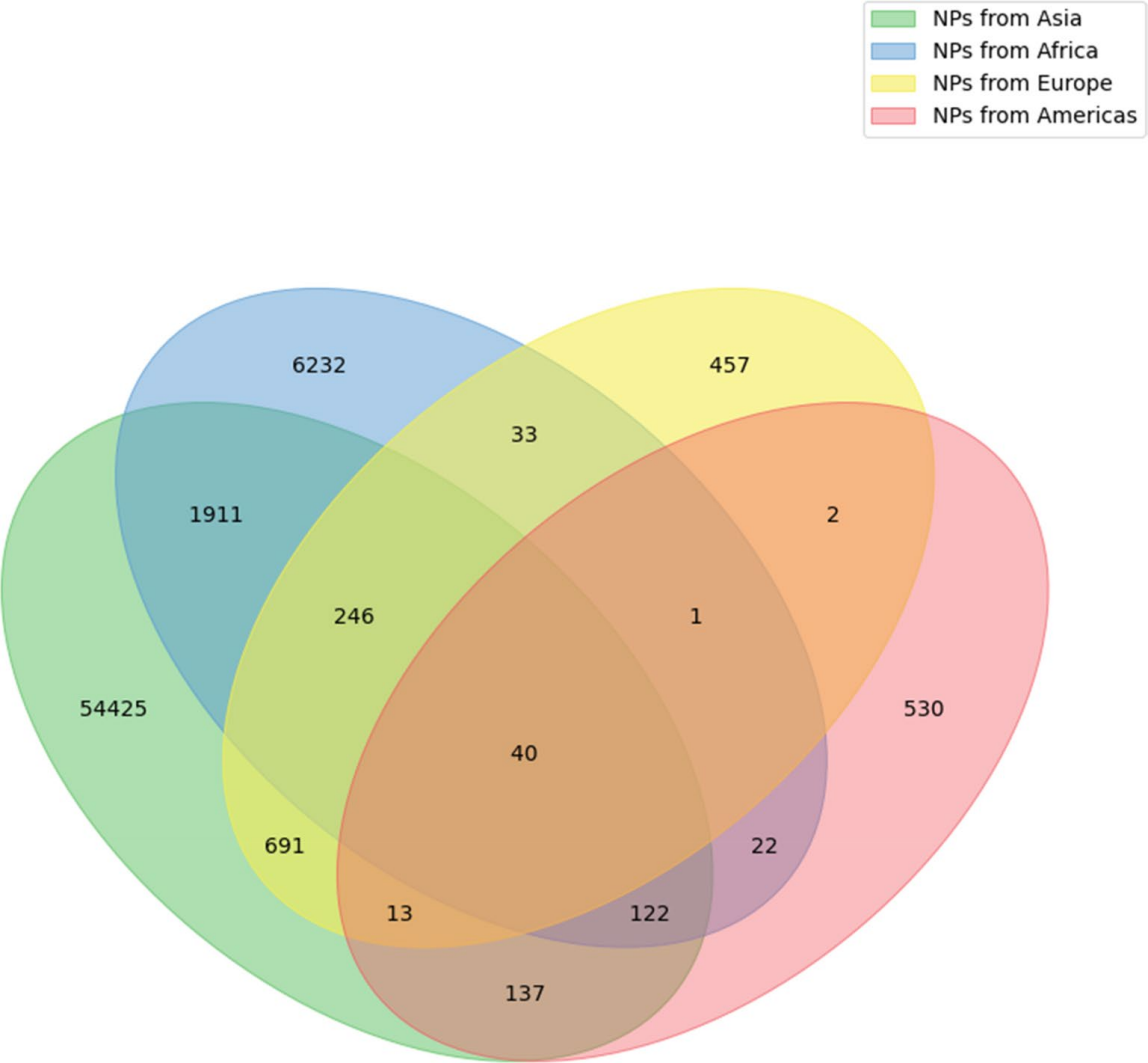
Overlap of NPs geographic provenance in COCONUT

For NPs where geographical information is available, it appears that most of them are produced by organisms that have been isolated in Asia (Fig. 4). This bias is introduced by the intensive study by scientists of the traditional Chinese and Indian medicines and by the big efforts in isolation and elucidation of NPs from medicinal plants. NPs from the African continent are also well represented in COCONUT (Fig. 4), mainly due to the scientific interest in African traditional medicines and African biodiversity. There is, for now, no data from the biodiversity of the Australian continent, and only very little data for NPs isolated from endemic European organisms. NPs from the Americas are mainly extracted and solved while Brazilian and Mexican biodiversity exploration. Only a few NPs are present in more than one continent, mainly in Asia and Africa, and the overlap values are biased by the very different NPs set sizes between the different continents.

### Web interface and technical specificities

All COCONUT data is stored with MongoDB, a cross-platform document-oriented NoSQL database program. The smallest unit in MongoDB is a document, composed of key and value pairs that are similar to JSON objects. Documents of the same nature are organized in collections, which are the equivalent of the SQL-based databases tables. MongoDB is particularly adapted to big and complex data, supports multiple indexing, including text indexing allowing enhanced text search in text-indexed fields and contains a wide range of in-build search and analysis functions.

Two major collections are present in the COCONUT database: SourceNaturalProduct, which contains the original NPs data collected from the open sources, and UniqueNaturalProduct, the unified and curated collection of NPs. The full version of COCONUT with all the calculated features can be accessed as a MongoDB dump in the Downloads section of the website. Requests for displaying additional crucial features in the web interface and making them searchable through the advanced search interface are welcome via the COCONUT GitHub tracker (see below).

The COCONUT online front-end is developed entirely with React.js [26], a JavaScript library to build responsive and efficient user interfaces. The OpenChemLib library [27] is used to handle the chemical editor for the search functions. The COCONUT back-end, allowing to process the front-end requests and to communicate with the database is written in Kotlin and Java 11 using the Spring framework. The CDK [13] library is used to process chemical information and formats.

COCONUT web interface, back-end and database are entirely Dockerised, allowing a quick and easy deployment on local servers and cloud. All the code, for both front-end and back-end, is available on GitHub (https://github.com/mSorok/NaturalProductsOnline).

### Searching the database

COCONUT online has been developed to be a full-fledged chemical database and in particular to fit the NPs structural and annotational particularities, with all the subsequent functions. At the moment, the chemical search is uncommon with MongoDB, therefore several approaches have been implemented to run molecular substructure and similarity searches.

#### Simple search

The so-called “simple” search can be performed using the header search bar. The users can enter there molecule names (e.g. “curcumin”), SMILES, InChI, InChi key, COCONUT ids and molecular formulas. Name search uses native MongoDB text indexing, allowing fuzzy, flexible search in the “name” and “synonyms” fields. The input string type is first identified using regular expressions, then the DB is queried against the appropriate fields, and the result, when exists, is returned to the front-end.

#### Substructure search implementation

Searching for an exact substructure in a MongoDB database of molecules appears to be surprisingly easy. Each molecule in the database needs to have their fingerprints of choice (in COCONUT are used the PubChem fingerprints) to be precomputed and stored as a list of bytes (BinData type in MongoDB). The query molecule (substructure) then needs to have its fingerprint to be also computed and to be matched against the database using the $allBitsSet function [28]. This native to MongoDB function allows to select documents in a collection where a BinData field has all the query bits set to “on” (but can have bits set to “on” that are not present in the query). To confirm the substructure match, the user can select between the default Ullmann [29], the Vento-Foggia [30] and the depth-first (DF) [31] pattern matching approaches, all performed using the CDK in-build algorithms. These three pattern matching techniques tend to, generally, return very similar results, the difference between them lying rather in their approach to matching substructures, therefore the usage of the default, Ullmann, method is to be privileged by users unfamiliar with the intricacies of such matching.

#### Similarity search implementation

Similarity search with MongoDB was implemented following the excellent ChEBML blog post on LSH-based similarity search in MongoDB [32] and adapted to Java, Kotlin and Spring data. In this approach, the MongoDB aggregation framework is used to perform inverted indexing search against PubChem fingerprints stored in a separate table and referencing COCONUT identifiers that contain the molecular features encoded by each bit.

#### Advanced search

The advanced search supports searching for NPs in COCONUT according to a range of parameters, such as molecular formula, molecular descriptor values, number of rings, type of sugar moieties present in them, etc.

#### Querying COCONUT through the API

A REST API has been developed for COCONUT online in order to permit programmatic querying and facilitate its integration in workflows. It relies on Kotlin API functionalities and it’s usage, together with some examples, is described in detail in the documentation section of the website (https://coconut.naturalproducts.net/documentation).

### Documentation

Complete documentation describing COCONUT, its data and functionalities are available at the documentation section of the website https://coconut.naturalproducts.net/documentation.

## Utility and discussion

The online COCONUT database is an open tool for researchers in the natural products community. COCONUT is the biggest collection of NPs in 2020 and the data it contains already benefits researchers in NPs with various aims, such as biodiversity research and drug discovery. The web interface allows querying and parsing the data collection in various, chemically relevant ways with adequate performance. It is also the first big chemical database using MongoDB as a storage management system.

A wide range of molecular descriptors are pre-computed and literature, producer taxonomy and their geography are as much annotated as currently possible without extensive manual curation. The web database can be searched in multiple ways, by molecular structure, by compound name and by molecular features, making this repository a complete chemical database. The user interface is modern and easy to use. Besides, the whole content of COCONUT is available for download in multiple formats.

In the close future, COCONUT will support user registration to enable user-driven NPs curation and submission and will undergo a better data annotation, in particular regarding the organisms that are producing the NPs, their geography and the corresponding literature, using deep learning approaches.

### Feedback

Bugs, annotation issues and requests of new COCONUT entries or re-annotation of existing ones can be reported at the project issues tracker (https://github.com/mSorok/NaturalProductsOnline/issues). Suggestions for new features are also welcome.

## Availability

All COCONUT data, code to process raw NPs data, data quality control and annotation, and the code for the font- and the back-end of the COCONUT online website are freely available without any restriction. The latest COCONUT data, as MongoDB full dump can be downloaded at https://coconut.naturalproducts.net/download. Code for data assembly, processing and quality control process codes is available on GitHub at https://github.com/mSorok/COCONUT. The code for the front-end and back-end is also available on GitHub at https://github.com/mSorok/NaturalProductsOnline.

## Conclusions

COCONUT is the largest open collection of elucidated and predicted NPs at this time. It has a great potential of being of particular importance for the NPs research community as it gathers most of open NPs knowledge in one single place, and makes it easily accessible and queryable.

The final aim of COCONUT is to provide to the scientific community NPs structures and their provenance, i.e.. organisms that synthesize them and geographic location of the latter. However, a lot of data curation, in particular using new generation deep learning-based methods of extracting information from publications and books, together with website functionalities developments are still need to be done for COCONUT, but the database as it is now is already an important tool to facilitate NPs and medicinal chemistry research.

## Data Availability

the source code of the web interface and the back-end is available on GitHub at https://github.com/mSorok/NaturalProductsOnline. The data was curated and processed using the COCONUT code suite available on GitHub at https://github.com/mSorok/COCONUT. All COCONUT data can be accessed on the website at https://coconut.naturalproducts.net/ and downloaded entirely or partially in several formats (MongoDB dump, SDF and SMI (SMILES)).

## Abbreviations

NPs: Natural products
COCONUT: COlleCtion of Open Natural ProdUcTs
CDK: Chemistry Development Kit
IUPAC: International Union of Pure and Applied Chemistry
API: Application Programming Interface
REST: REpresentational State Transfer
DOI: Digital Object Identifier
JSON: JavaScript Object Notation
(No)SQL: (non-/not only) Structured Query Language
